# Noble Gas Bonding Interactions Involving Xenon Oxides and Fluorides

**DOI:** 10.3390/molecules25153419

**Published:** 2020-07-28

**Authors:** Antonio Frontera

**Affiliations:** Department of Chemistry, Universitat de les Illes Balears, Crta de valldemossa km 7.5, 07122 Palma de Mallorca (Baleares), Spain; toni.frontera@uib.es

**Keywords:** noble gas interactions, noncovalent interactions, crystal packing, xenon

## Abstract

Noble gas (or aerogen) bond (NgB) can be outlined as the *attractive interaction between an electron-rich atom or group of atoms and any element of Group-18 acting as an electron acceptor*. The IUPAC already recommended systematic nomenclature for the interactions of groups 17 and 16 (halogen and chalcogen bonds, respectively). Investigations dealing with noncovalent interactions involving main group elements (acting as Lewis acids) have rapidly grown in recent years. They are becoming acting players in essential fields such as crystal engineering, supramolecular chemistry, and catalysis. For obvious reasons, the works devoted to the study of noncovalent Ng-bonding interactions are significantly less abundant than halogen, chalcogen, pnictogen, and tetrel bonding. Nevertheless, in this short review, relevant theoretical and experimental investigations on noncovalent interactions involving Xenon are emphasized. Several theoretical works have described the physical nature of NgB and their interplay with other noncovalent interactions, which are discussed herein. Moreover, exploring the Cambridge Structural Database (CSD) and Inorganic Crystal Structure Database (ICSD), it is demonstrated that NgB interactions are crucial in governing the X-ray packing of xenon derivatives. Concretely, special attention is given to xenon fluorides and xenon oxides, since they exhibit a strong tendency to establish NgBs.

## 1. Introduction

Molecular recognition and self-assembly are concepts related to supramolecular chemistry [1,2,3,4,5], where molecules interact with other molecules or themselves. These processes are guided by noncovalent interactions that spontaneously govern the formation of supramolecular assemblies. Chemists working in this field of research desire to control the molecular recognition process as precisely as possible, to generate more effective receptors, polymers, sensors, catalysts, etc. 

**Figure 1:**
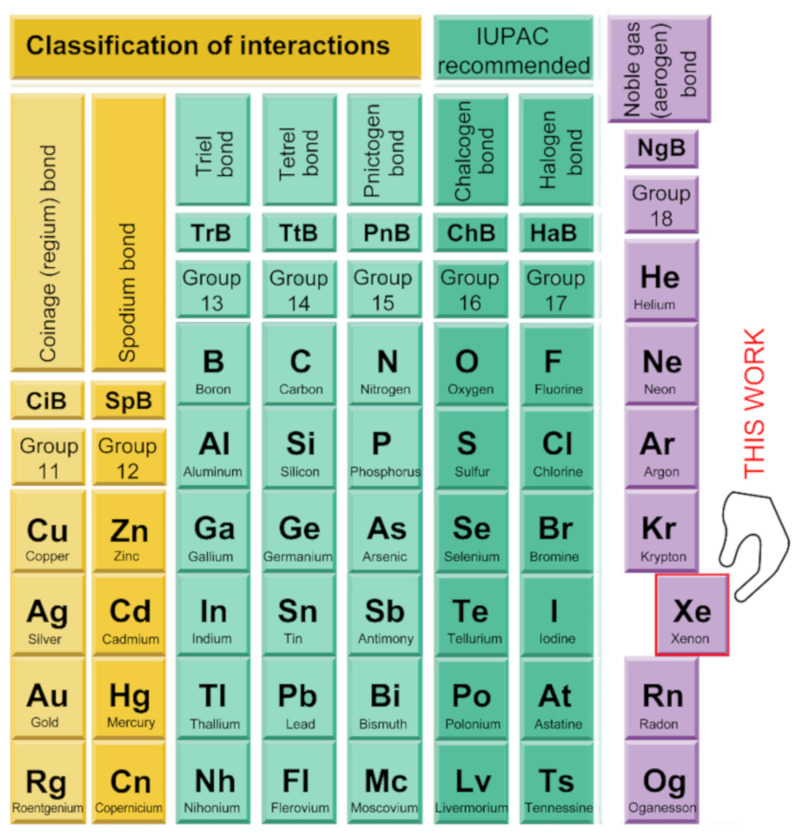
Groups 11 to 18 and their respective names that are used in the literature.

Crystal engineers and supramolecular chemists need to deeply understand the physical nature of noncovalent interactions and their distinctive properties such as strength, tunability, directionality etc., for the successful control of supramolecular chemistry processes [6]. Such control is a complicated task because these processes are usually governed by an intricate combination of noncovalent interactions [6,7]. The strength and strong directionality of hydrogen and halogen bonds (HB and HaB, respectively) make them ideal for their use in crystal engineering and molecular recognition. They are responsible for the significant progress in both fields during the last decades [8,9,10]. Furthermore, interactions involving aromatic rings (π–π, C–H⋯π, lone pair–π, ion–π) are also commonly used in both fields [11,12,13,14] being more relevant those involving electron-rich and/or electron-poor π-surfaces, since they exhibit stronger interaction energies. Moreover, other more unconventional interactions are gaining the interest of the scientific community and are increasingly taken into consideration for the construction of molecular receptors and catalysts [15,16,17,18,19,20]. These interactions can be divided into σ–hole [21,22,23,24] and π–hole interactions [25,26,27,28,29] and are nowadays entered into the toolkit supramolecular chemists. Actually, tetrel (Tt) [30,31,32,33,34,35,36,37,38], pnictogen (Pn) [39,40,41,42,43,44,45,46,47,48,49], and chalcogen (Ch) [50,51,52,53,54,55,56,57,58,59,60,61,62,63,64,65,66,67,68] bonding interactions (see Figure 1) have been studied by many theoretical works and are progressively used experimentally in relevant fields such as supramolecular catalysis, polymers, transmembrane ion transport and, especially, crystal engineering [15,16,17,18,19,20,21,22,23,24,25,26,27,28,29,30,31,32,33,34,35,36,37,38,39,40,41,42,43,44,45,46,47,48,49]. The general features of π,σ–hole interactions are as follows: (1) the strength of the interaction depends on the polarizability of the π,σ–hole donor atom (Lewis acid), (2) the electron-withdrawing ability of atoms directly bonded to it, and (3) the basicity of the electron-rich atom (π,σ–hole acceptor atom), usually a lone pair or a π–system. Several theoretical works have evidenced that noble gas (or aerogen) bonds (NgBs) behave parallel to those of elements of groups 13 to 17 [22,23]. Thus, a more positive π,σ–hole is expected for xenon derivatives and especially those compounds where the xenon is bonded to the most electronegative elements of the periodic table, fluorine, and oxygen.

In the late 1970s, gas phase Ng halides and oxides motivated active interest for their application in the UV laser action and for the characterization of their role in many other gas-phase elementary processes [69,70,71,72]. Moreover, the investigation of the nature of involved interactions has been also an important target of the basic research [73,74,75,76,77]. 

Gas phase Ng halides and oxides have permitted to define the role of half-filled atomic orbital alignment within the interacting adducts and to map the transition from pure noncovalent (van der Waals) to one-electron chemical bonds [78,79]. This transition has been described taking into account properly the role of charge (electron) transfer contribution to the formed bond [80].

Noble gas or aerogen-bonding interactions were termed as such in 2015 [81], and afterward, several experimental [82,83,84] and theoretical [85,86,87,88,89] investigations have appeared in the literature describing and confirming their relevance in X-ray structures and also its interplay with other interactions. The purpose of this short review is to examine the recent research on NgB interactions, including theoretical and experimental investigations. The physical nature of the interaction and its mutual influence with other forces is described in the first place. Moreover, selected and relevant X-ray solid-state crystal structures retrieved from the Cambridge Structural Database (CSD) and the Inorganic Crystal Structural Database (ICSD) are described and discussed. This second part is divided into two subsections, xenon fluorides (XeF_2_, XeF_4_, and XeF_6_) and xenon trioxide, since they present different characteristics (directionality and strength). Moreover, the noble gas bonds are σ–hole based interactions in XeF_6_ and XeO_3_ adducts and π–hole based interactions in XeF_2_ and XeF_4_ adducts.

## 2. Results

### 2.1. Pioneering Works and Physical Insights

**Figure 2:**
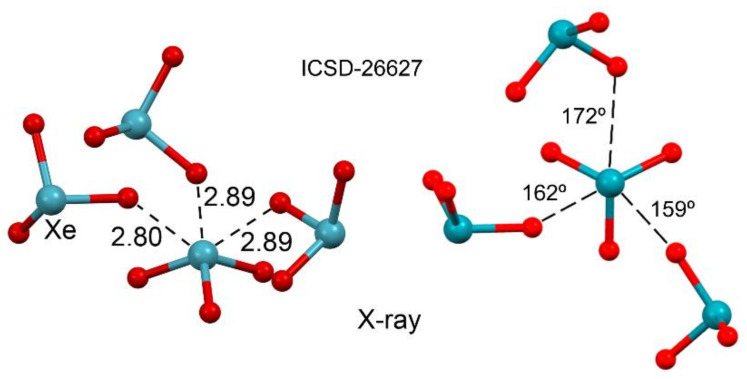
Ball and Stick representation of the X-ray structure (two views) of XeO_3_, refcode ICSD-26627. Distances in Å.

As aforementioned, in 2015, the term aerogen-bonding interaction was coined [81] to define the attractive interaction between elements of groups 18 acting as Lewis acids and any electron-rich atom or group of atoms. That work was inspired by the X-ray structure of XeO_3_ that was characterized by single-crystal X-ray diffraction in 1963 at room temperature (see Figure 2) [90]. More recently, the low-temperature X-ray crystal structure of XeO_3_ has been reported, revealing the existence of two new phases [91], and remarkably, in all three polymorphs, XeO_3_ establishes three Xe⋯O NgBs bonds, resulting in extended networks. The NgB distances of the crystalline phase shown in Figure 2 (at room temperature) are longer than the sum of covalent radii (2.06 Å) and shorter than the sum of van der Waals radii (3.68 Å), thus indicating strong interactions. Moreover, the noncovalent contacts are also quite directional (Figure 2, right), where the electron-rich O atom is located opposite to the O=Xe bonds, as typical in σ–hole interactions.

The behavior of XeO_3_ in the solid state has also been studied theoretically to analyze the effect of pressure (up to 50 GPa) [92]. Quite remarkably, the covalent Ng–O bond lengthens upon increasing the pressure, and at approximately 2 GPa, the O atom can move between both Xe atoms that form the Xe–O⋯Xe NgB similarly to low barrier H-bonds. The theoretical study suggests that this movement is responsible for the phase transition from *P*_212121_ to *P*_nma_ structure. 

**Figure 3:**
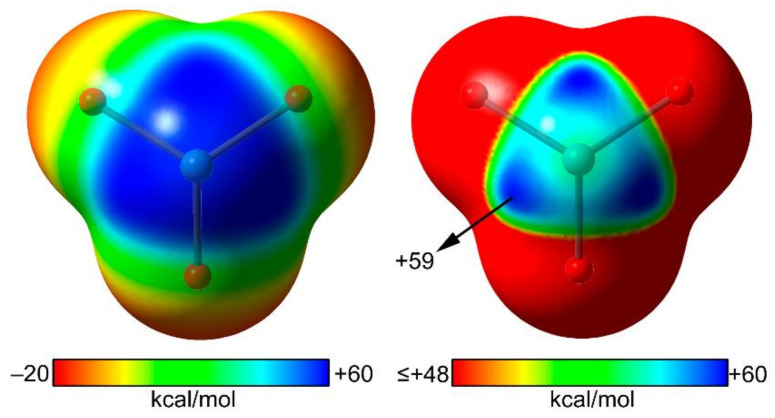
Molecular electrostatic potential (MEP) surfaces of XeO_3_ using two different scales. Isosurface 0.01 a.u.

Figure 3 shows the molecular electrostatic potential (MEP) surface of XeO_3_ at the MP2/aug-cc-pVQZ level of theory with a large region of positive potential that covers the location of the stereo active lone pair at the Xe atom. The value of MEP at the σ–hole is comparable to those reported for heavy pnicogen, chalcogen, and halogen atoms [22]. The atomic polarizability of xenon (27.1 a.u.) is comparable to those reported for other elements of row 5, for instance, 32.3 a.u. for I and 38.3 a.u. for Te. The MEP surface reveals that the anisotropy at the Xe atom is not evident, in contrast to the behavior of heavy halogen and chalcogen atoms, but similar to pnicogen atoms [22]. Figure 3 also shows the MEP surface using a narrower scale that reveals three symmetrically distributed σ–holes at the extensions of the O=Xe bonds, thus explaining the directionality of the NgBs shown in Figure 2.

The strengths of several NgBs taken from the literature are gathered in Table 1 [81]. They correspond to complexes of XeO_3_ with two Lewis bases and two anions. The calculated values show that interaction energies with neutral Lewis bases are greater than conventional H-bonds (for instance, the H-bond in the water dimer is around 5 kcal/mol). NgBs are very strong in the complexes with anions (Cl^−^ and Br^−^) due to the dominance of electrostatic effects.

**Figure 4:**
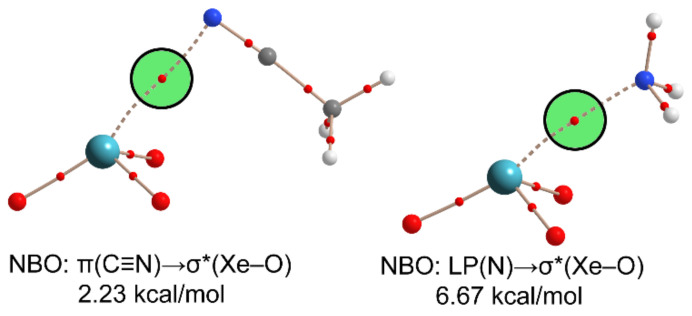
Distribution of bond critical points (in red) and bond paths in the XeO_3_⋯NCCH_3_ (**left**) and XeO_3_⋯NH_3_ (**right**) complex. The results from the Natural Bond Orbital (NBO) analysis are also indicated close to the complexes. LP stands for lone pair.

The NgB interaction was also characterized by the quantum theory of atoms in molecules (QTAIM) [81]. The QTAIM analyses of the neutral complexes are shown in Figure 4 with the typical bond critical point (represented by a small red sphere) and bond path (noncovalent bond paths represented as dashed lines) connecting the Xe atom to the electron-rich N atom. Moreover, the importance of orbital contributions in NgBs was studied using Natural Bond Orbital (NBO) calculations [93]. It is well known that σ–hole interactions are characterized by a typical orbital donor–acceptor interaction where a lone pair (LP) orbital (Lewis base) or π orbital (π–system as electron-donor) donates electron density to an antibonding σ* orbital of the σ–hole donor atom. For the XeO_3_⋯NCCH_3_ complex (Figure 4, left), orbital effects are moderate (approximately 22% of the total interaction energy of Table 1), and interestingly, the donor orbital is the nitrile’s π–system. For the XeO_3_⋯NH_3_ complex, the orbital contribution is significant (> 50%), and the donor orbital is the N-lone pair (see Figure 4, right).

**Figure 5:**
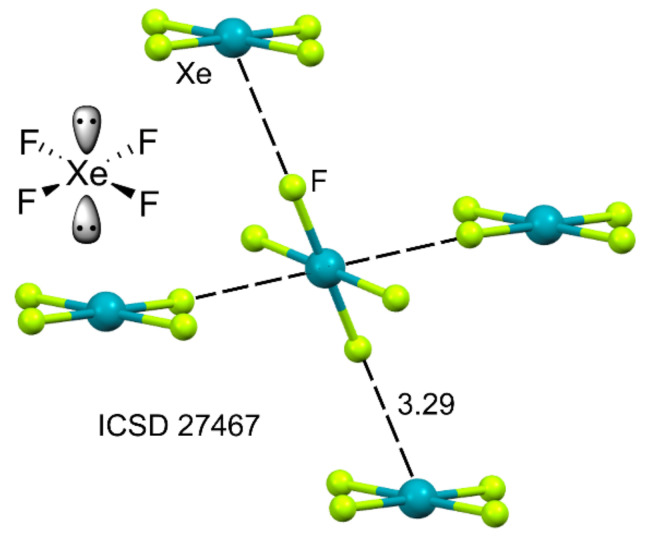
Ball and Stick representation of the X-ray structure of XeF_4_, refcode ICSD-27467. Distance in Å.

The π–hole version of the NgBs was also described in 2015 using XeF_4_ as a prototypical molecule [94]. This molecule is square planar, facilitating the approximation of Lewis bases above and below the molecular plane. In the solid-state structure of xenon tetrafluoride (see Figure 5), each molecule establishes four symmetrically related NgB contacts, two as donor and two as acceptor at an Xe⋯F distance that is longer than the sum of covalent radii (1.97 Å) and shorter than the sum of van der Waals radii (3.63 Å). The approximation of the F atom of the adjacent molecule toward the Xe atom is not precisely perpendicular. This is due to the presence of two stereoactive lone pairs above and below the Xe(IV) atom (see Figure 5).

**Figure 6:**
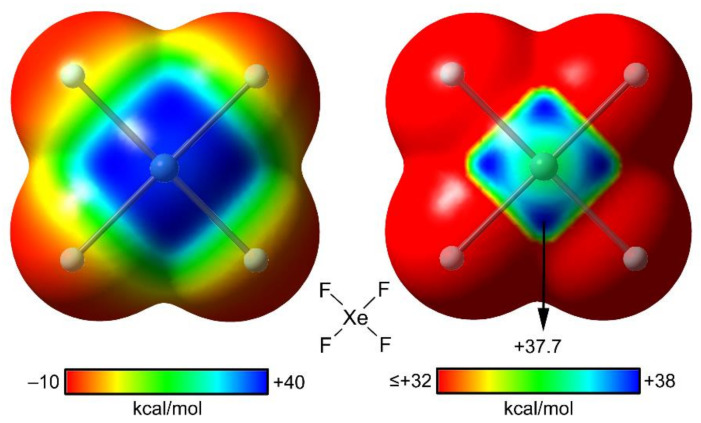
MEP surfaces of XeF_4_ using two different scales shown below the surfaces. Blue color is used for the maximum and red color for the minimum MEP value. Isosurface 0.01 a.u.

The MEP surfaces of XeF_4_ using two different energetic scales are shown in Figure 6. The π–hole occupies the major part of the molecular plane, including the lone pairs (LPs) of the Xe(IV) that are located above and below the Xe atom along the main symmetry axis. The stereo active LPs’ effect is revealed when a narrower scale is used (see Figure 6, right), where four symmetrically equivalent MEP maxima appear. They are located along the bisectors of the F–Xe–F angles. This distribution of the molecular charge around the Xe atom explains the directionality of the NgBs in the X-ray structure of XeF_4_ (see Figure 5).

**Figure 7:**
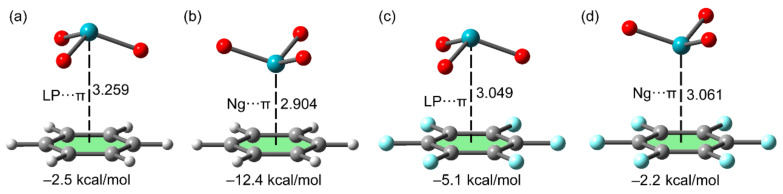
Ng⋯π and lone pair⋯π complexes of XeO_3_ with benzene (**a**,**b**) and hexafluorobenzene (**c**,**d**) and their interaction energy. Distances in Å.

NgB complexes where aromatic rings act as electron donors have also been analyzed theoretically [95] and compared to lone pair⋯π interactions. Figure 7 shows two different binding modes of XeO_3_ interacting with electron-rich and electron-poor π-systems: benzene and hexafluorobenzene, respectively. The energetic results gathered in Figure 7 reveal that the Ng–π interaction established between the XeO_3_ molecule and the C_6_H_6_ ring is the strongest of the series (−12.4 kcal/mol). Conversely, the lone pair⋯π interaction (the O atoms instead of the σ–hole of the Xe atom point to the aromatic ring) presents the largest interaction for the C_6_F_6_ ring (−5.1 kcal/mol). Similar results have been described for XeF_4_ [95] interacting with electron-rich and electron-poor aromatic rings. Miao and Song [96] have demonstrated using state-of-the-art ab initio calculations that the electrostatic and dispersion forces are dominant in Ng–π interactions (around 50% of the total contribution) followed by the induction (13 %). Ebrahimi et al. [97] have also studied Ng⋯π interactions in a series of six π-electron pseudoaromatic heterocyclic compounds, including boraphosphinine, borazine, and alumazine, demonstrating that dispersion plays an important role in the binding mechanism.

Gao et al. [98] in 2016 analyzed theoretically Ng··π interactions in a variety of systems (HC≡CH, H_2_C=CH_2_, C_6_H_6_, and C_4_H_4_X, X = NH, O and S) using XeOF_2_ as the Lewis acid. They evidenced that the strength of the Ng–π interaction is comparable to hydrogen bonds. Using the same xenon derivative, Scheiner et al. [99] studied theoretically the energetic features of its complexes with diazines showing stabilization energy up to 18 kcal/mol due to the existence of ancillary CH⋯F contacts involving the aromatic C–H groups, although the main contributor is the NgB. 

Esrafili et al. [100] have used xenon trioxide as an σ–hole donor to analyze substituents effects in RC≡N and H_2_NR Lewis bases. The interaction energy can be easily tuned by the R substituent and ranges from −4.5 to −13.6 kcal/mol depending on the electron donating/withdrawing ability of R and the hybridization of the N atom. In this type of complex, the LP(N)→σ*(Xe-O) contributions are significant, and the ^131^Xe–NMR chemical shift value decreases upon complexation. Besides, the same research group analyzed the interaction of xenon trioxide with radical donors [101]. They showed that single-electron NgBs are energetically favorable. Interestingly, the simultaneous formation of HBs enhances the strength of the single-electron NgB, thus revealing cooperativity effects. 

The energetic and geometric features of complexes of xenon trioxide with anions (CN^−^, halides and pseudohalides) have also been studied utilizing ab initio calculations [102]. The binding energies are considerable (approximately −65 kcal/mol), and the NgB exhibits partial covalency. Furthermore, the interaction of carbene species with xenon trioxide has been reported [103], where the carbene acts as an electron-rich molecule. Finally, the ability of XeO_3_ to form bifurcated NgBs with catechol derivatives has been investigated [104] with interaction energies as large as −15.6 kcal/mol. Noble gas bonds with chalcogen donors including an interesting analysis of substituent effects have been recently reported by Scheiner et al. [105]. The influence of the size of the chalcogen atom upon the binding energies was also investigated.

**Figure 8:**
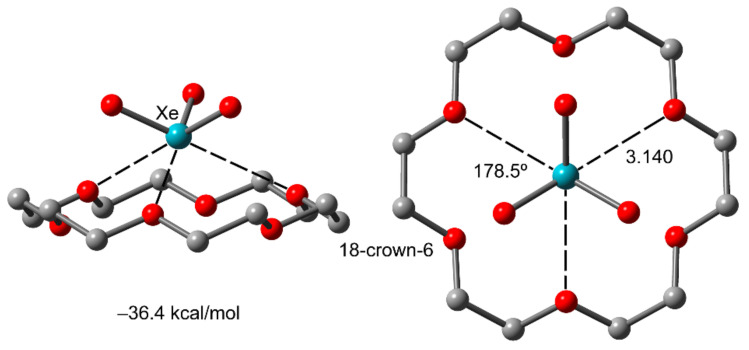
Perspective and on-top views of the complex of XeO_3_ with 18-crown-6 and its interaction energy. Distances in Å.

It is worthwhile to comment on the extraordinary strong binding energy found for the supramolecular complex of XeO_3_ with 18-crown-6 receptor (see Figure 8) by Miao et al. [106]. This extremely strong binding force (36.4 kcal/mol) is comparable to the strong cation–π interaction. It is due to the concurrent formation of three highly directional NgBs (178.5°) with three O atoms of the 18-crown-6 receptor.

### 2.2. Cooperativity 

#### 2.2.1. NgBs and H-bonds or Alkali (Lithium) Bonds

**Figure 9:**
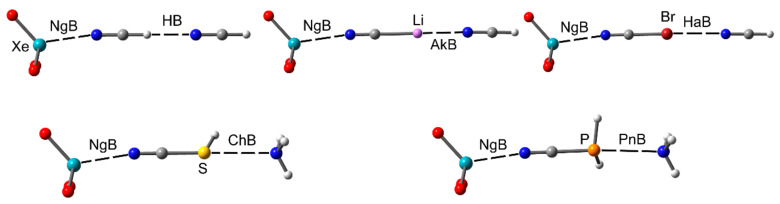
Ternary systems used by Esrafili et al. [107,108,109] to study the cooperativity effect between noble gas (or aerogen) bond (NgBs) and AkB, HaB, ChB, and PnB interactions using XeO_3_ as an NgB donor.

Vessally et al. in 2016 [107] analyzed and demonstrated synergistic effects in ternary systems (see Figure 9) of the general formula O_3_Xe⋯NCH⋯NCR and O_3_Xe⋯NCLi⋯NCR (R=H, F and CH_3_), using several criteria based on cooperativity energies and the variation of the equilibrium distances. A shortening of both noncovalent distances (NgB and either HB or alkali bond) is observed in the ternary complexes compared to the binary ones. Synergistic effects were more prominent in those complexes where alkali and noble gas bonds coexist.

#### 2.2.2. NgBs and ChB and PnB Interactions

Cooperativity effects between an NgB and ChB or PnB interactions have been investigated in ternary H_3_N⋯PH_2_CN⋯XeO_3_ and H_3_N⋯SHCN⋯XeO_3_ assemblies, among others (see Figure 9, bottom) by Esrafili et al. using high-level ab initio calculations [108] and taking into account solvent effects. For both combinations of interactions ChB/NgB and PnB/NgB, a favorable interplay between the interactions was demonstrated with a mutual reinforcement of both. This effect was more relevant in the PnB/NgB complexes than ChB/NgB ones. The reinforcement of the NgB was more significant than either the PnB or ChB in the ternary complexes.

#### 2.2.3. NgB and HaB Interactions

Esrafili et al. [109] have also investigated the geometric and energetic features of ternary complexes O_3_Xe⋯NCX⋯NCY (X = Cl, Br, I and Y = H, F, OH) using high-level ab initio calculations, see Figure 9 (top, right) for a representation of the O_3_Xe⋯NCBr⋯NCH assembly. Both intermolecular interactions present in these complexes, NgB and HaB, shorten in the trimer compared to the isolated dimers, thus suggesting a mutual reinforcement. Moreover, the existence of synergetic effects was demonstrated and rationalized using MEP, quantum theory of atoms in molecules (QTAIM) and noncovalent interaction index (NCIPlot) computational tools. Further evidence for the existence of cooperativity effects was obtained by computing the spin–spin coupling constants across the NgB bonding, *J*(Xe–N), which decreases in the ternary complex with respect to the binary complex.

#### 2.2.4. NgBs and Anion⋯π or Lone Pair⋯π Interactions

**Figure 10:**
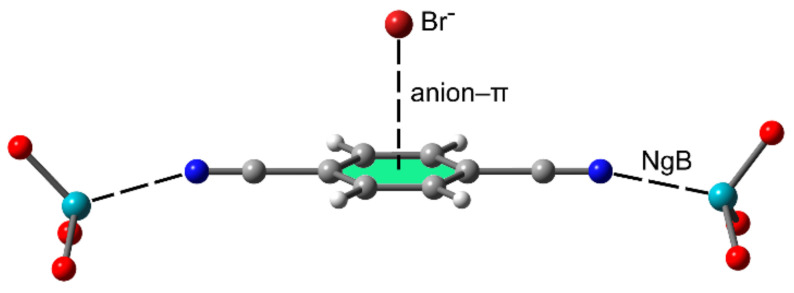
Four component system used by Esrafili et al. [97] to study the cooperativity effect between NgBs and anion–π interactions using XeO_3_ as an NgB donor and 1,4-dicyanobenzene.

Cooperativity effects between NgB and anion/lone pair–π interactions have also been studied [110] using 1,4-dicyanobenzene as a π-acidic ring (see Figure 10 for a representative example). In addition to the ability of 1,4-dicyanobenzene to establish anion/lone pair⋯π interactions, it can also participate in NgBs as an acceptor (weak Lewis base) via the sp-hybridized lone pair at the N atom. The formation of two NgBs with XeO_3_ increases the π acidity of the aromatic ring significantly, thus reinforcing the anion/lone pair⋯π interaction. The cooperativity energies are large for the combination NgB/anion⋯π with values up to −12 kcal/mol and more modest (around 1 kcal/mol) for the combination NgB/lone pair⋯π.

### 2.3. NgB in XeO_3_ Adducts

**Figure 11:**
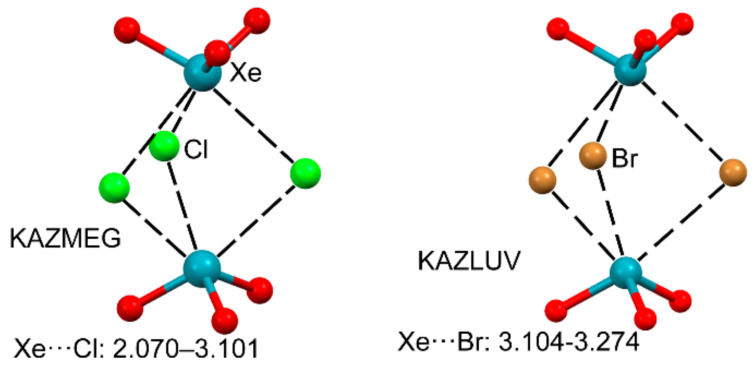
Partial views of the X-ray structures corresponding to Cambride structural database (CSD) reference codes KAZMEG (**left**) and KAZLUV (**right**) corresponding to tris(tetraethylammonium), tris(μ-chloro)-(μ-oxo)-octaoxo-tri-xenon, and tris(tetraethylammonium), tris(μ-bromo)-(μ-oxo)-octaoxo-tri-xenon acetonitrile solvate, respectively. Distances in Å. Counter-cations and solvent molecules omitted for clarity.

As discussed above, the XeO_3_ molecule presents a large and intense region of positive potential at the lone pair location and three σ–holes located opposite the Xe=O bonds. Therefore, it exhibits a strong tendency to form directional noncovalent interactions as exemplified by its X–ray solid state structure represented in Figure 2. Several investigations by Schrobilgen’s group have further demonstrated the ability of XeO_3_ to act as Lewis acid and have evidenced the importance of NgBs in the solid state of several adducts [111,112,113]. For instance, halide salts of general formulae [N(C_2_H_5_)_4_]_3_[X_3_(XeO_3_)_3_]; X = Cl, Br were synthesized and X-ray characterized (see Figure 11) at low temperature [98]. Each XeO_3_ molecule establishes three directional NgBs where the halides are located opposite to the Xe=O bonds in agreement with the location of the three σ–holes represented in Figure 3. It is worthy of highlighting that the KAZLUV structure (Figure 11, right) is the first and unique example of a Xe⋯Br NgB reported in the literature.

**Figure 12:**
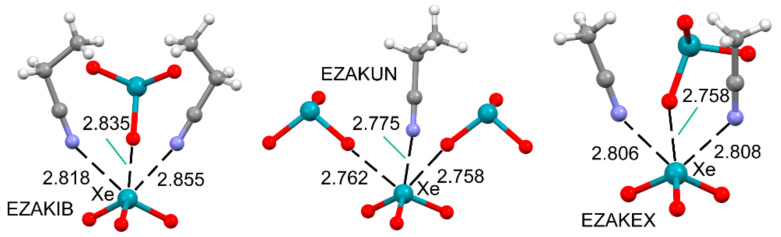
Partial views of the X-ray structures corresponding to CSD reference codes EZAKIB (**left**), EZAKUN (**middle**), and EZAKEX (**right**) corresponding to bis(propionitrile)-trioxo-xenon, trioxo-propanenitrile-xenon, and bis(acetonitrile)-trioxo-xenon, respectively. Distances in Å.

The predisposition of xenon trioxide to establish three simultaneous NgBs was corroborated by Schrobilgen’s group [82] in a series of XeO_3_·RCN adducts (R = Me or Et). Three adducts are represented in Figure 12, and all of them present three NgBs that direct their crystal packing. In EZAKIB and EZAKEX, the Xe establishes two Xe⋯N NgBs with the RCN molecule and one additional Xe⋯O NgB with an adjacent XeO_3_ molecule, while in the EZAKUN structure, the Xe establishes one Xe⋯N and two Xe⋯O NgBs.

**Figure 13:**
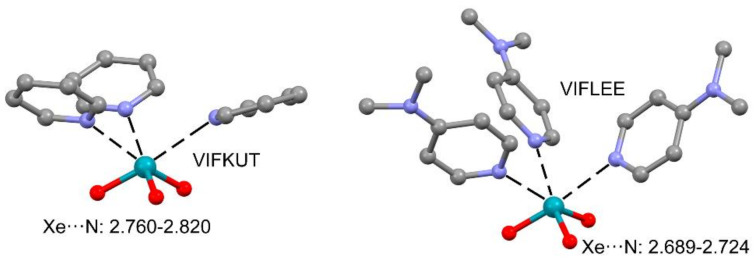
Partial views of the X-ray structures corresponding to CSD reference codes VIFKUT (**left**) and VIFLEE (**right**). Distances in Å. The H-atoms are omitted for clarity.

The same research group has also reported two additional X-ray structures where the electron donor atom is the sp^2^-hybridized N-atom of pyridine (see Figure 13). The intermolecular Xe⋯N distances in the VIFKUT structure are longer than those in VIFLEE [112] because the presence of the dimethylamino group in *para* increases the basicity at the N-atom, thus enhancing the NgB.

**Figure 14:**
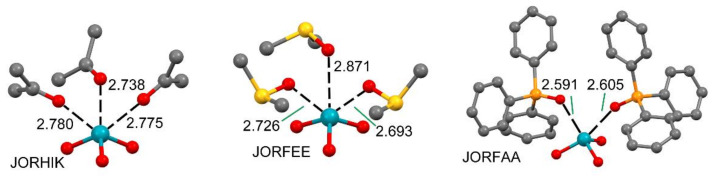
Partial views of the X-ray structures corresponding to CSD reference codes JORHIK (**left**), JORFEE (**middle**), and JORFAA (**right**) corresponding to bis(acetone)-trioxo-xenon acetone-trioxo-xenon acetone solvate, (μ-dimethyl sulfoxide)-bis(dimethyl sulfoxide)-hexaoxo-di-xenon, and bis((triphenyl)phosphine oxide)-trioxo-xenon, respectively. Distances in Å. The H atoms are omitted for clarity.

The low-temperature, single-crystal X-ray characterization of XeO_3_ adducts with triphenylphosphine oxide, dimethylsulfoxide, pyridine-N-oxide, and acetone by the formation of NgBs has been recently reported (three of them represented in Figure 14) [113]. These interactions give stability to the otherwise easy to detonate XeO_3_ solid. In acetone and DMSO adducts (JORHIK and JORFEE, respectively), the XeO_3_ establishes three Xe⋯O σ-hole interactions. However, the JORFAA structure only shows two Xe⋯O NgBs, which is quite uncommon in XeO_3_ adducts and can be attributed to steric effects due to the presence of the phenyl rings. 

### 2.4. NgB in XeF_2_, XeF_4_, and XeF_6_

**Figure 15:**
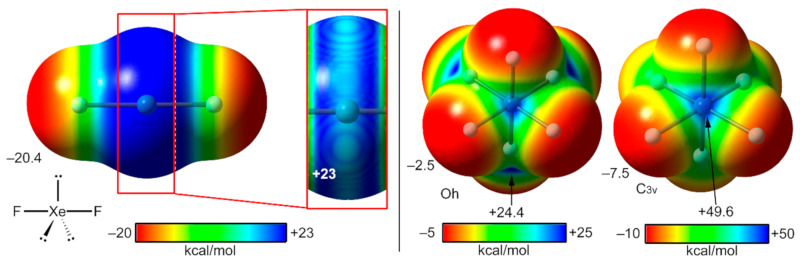
**Left**: MEP surface of XeF_2_ showing the location of the positive belts. Isosurface 0.01 a.u. **Right**: MEP surfaces of the octahedral and *C*_3v_ forms of XeF_6_.

The molecular electrostatic potential (MEP) surface of XeF_4_ has been commented on above (see Figure 6). Figure 15 shows the MEP surfaces of XeF_2_ and XeF_6_ [88] that are worthy of mentioning, before the analysis of their X-ray structures. The MEP surface of XeF_2_ shows a positive belt around the Xe atom and two symmetrically equivalent negative regions at the F atoms. A close examination of the surface reveals that the maximum positive belt is not exactly perpendicular to the molecular axis at the Xe atom position. Instead, the MEP surface shows two maximum belts (+23 kcal/mol) that are slightly displaced toward the F-atoms. This is likely due to the effect of the three stereo-active lone pairs of the Xe atom. Therefore, it is expected that the Ng-bonding interactions with Lewis bases will likely present some deviation from the perpendicular trajectory. 

The MEP surface of xenon hexafluoride is also represented in Figure 15 (right panel) using two different symmetries: octahedral and *C*_3v_. For the octahedral XeF_6_, eight equivalent and moderately strong (+24.4 kcal/mol) σ–holes appear in the middle of each face of the octahedral. For the *C*_3v_ form, a unique σ–hole with a very large MEP value is found (+49.6 kcal/mol) located at one face of the polyhedron along the *C*_3_ axis. The global minimum structure of XeF_6_ is still under investigation [114,115,116]. Most of the theoretical works suggest that the O_h_ form is the most stable, which is in disagreement with experimental findings that indicate that the *C*_3v_ isomer is more stable (it is the one observed in X-ray structures). State of the art calculations propose that both forms are approximately isoenergetic [117], thus suggesting that this molecule is highly fluxional. 

#### 2.4.1. X-Ray Structures of XeF_2_

**Figure 16:**
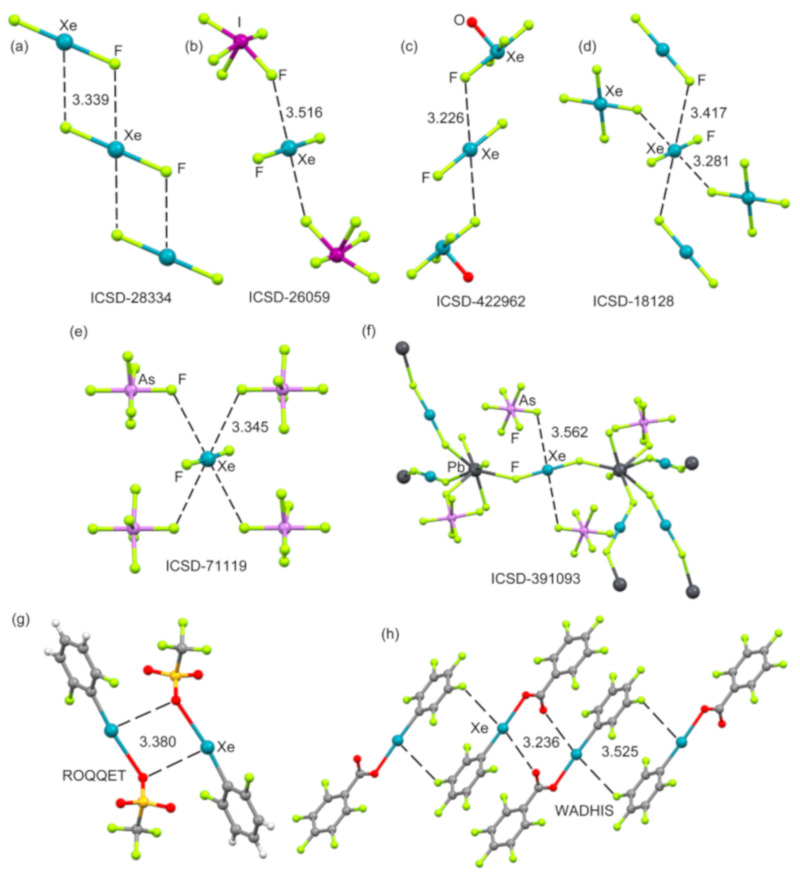
Partial views of the X-ray structures corresponding to ICSD and CSD reference codes ICSD-28334 (**a**), ICSD-26059 (**b**), ICSD-422962 (**c**), ICSD-18128 (**d**), ICSD-71119 (**e**), ICSD-391093 (**f**), ROQQET (**g**), and WADHIS (**h**), corresponding to xenon difluoride, xenon difluoride-iodine pentafluoride, xenon difluoride-xenon oxytetrafluoride, xenon difluoride-xenon tetrafluoride, Ag(XeF_2_)_2_(AsF_6_), Pb(XeF_2_)_3_(AsF_6_)_2_, 2,6-difluorophenyl-xenon trifluoromethanesulfonate, and pentafluorobenzoato-pentafluorophenyl-xenon, respectively. Distances in Å.

The ICSD contains several X-ray structures including the XeF_2_ molecule in their structure, which are represented in Figure 16. The distances of the NgB contacts are in all X-ray structures longer than the sum of covalent radii and shorter than the van der Waals (Xe + F = 3.63 Å), thus confirming the noncovalent nature of these interactions. Regarding their directionality, all structures present F–Xe⋯F angles that are smaller than 90°, which is in agreement with the MEP surface commented above (see Figure 15, left). The ICSD-28334 reference code corresponds to the XeF_2_ crystal structure [118], which forms 1D supramolecular polymers in the solid state governed by the formation of two symmetrically equivalent Xe⋯F contacts (see Figure 16a). The assemblies observed in the co-crystals [XeF_2_]·[IF_5_] and [XeF_2_]·[XeF_4_O] represented in Figure 16b,c are comparable since in both, one equatorial F-atom of the IF_5_ or XeF_4_O molecules points to the Xe atom of the adjacent XeF_2_ molecule (see Figure 16a,b). The ICSD-18128 reference code corresponds to the XeF_2_·XeF_4_ co-crystal where the central the Xe atom establishes four Xe⋯F contacts with both XeF_4_ and XeF_2_ neighboring molecules (see Figure 16d).

An interesting aspect of the XeF_2_ molecule is its role as a ligand to synthesize coordination compounds combined with elements of groups I and II, transition metals, lanthanides, and lead [119]. There are several reviews in the literature describing coordination compounds with XeF_2_ as a ligand [120,121]. Figure 16e,f shows two selected examples of coordination compounds where the XeF_2_ molecules establish two symmetrically equivalent NgBs. In the ICSD-71119 structure, the F atoms are coordinated to Ag ions, which are not shown for clarity. It can be observed that the XeF_2_ molecule establishes four Xe⋯F contacts with the counter-anions [122]. It is expected that the coordination of XeF_2_ to the metal center enhances the ability of Xe to act as Lewis acid. The ICSD-391093 (Figure 16f) corresponds to a coordination polymer where each XeF_2_ molecule is coordinated to two Pb metal centers [123]. Both symmetrically equivalent Xe⋯F contacts are formed with the AsF_6_^−^ counterions.

Figure 16g,h shows two additional X-ray structures [124,125] exhibiting Xe⋯O/F contacts retrieved from the CSD, where the xenon(II) is linear. Although both X-ray structures do not correspond to XeF_2_ derivatives, they are worthy of commenting to further evidence of the ability of linear Xe(II) compounds to establish directional NgBs in the solid state.

#### 2.4.2. X-Ray Structures of XeF_4_

**Figure 17:**
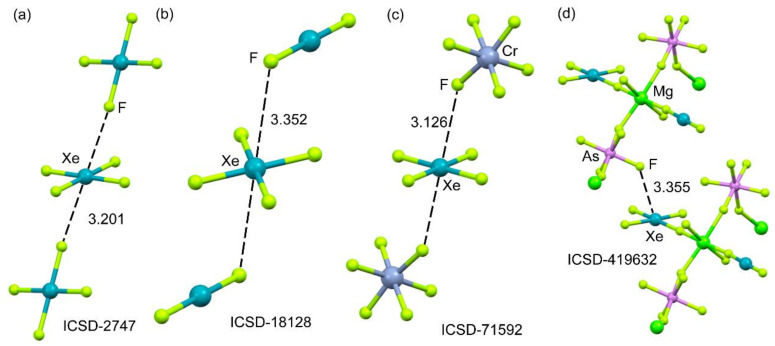
Partial views of the X-ray structures corresponding to ICSD reference codes ICSD-2747 (**a**), ICSD-18128 (**b**), ICSD-71592 (**c**), and ICSD-419632 (**d**), corresponding to xenon tetrafluoride, xenon difluoride-xenon tetrafluoride, (CrF_6_)_4_(XeF_4_)_5_ and [Mg(XeF_2_)(XeF_4_)](AsF_6_)_2_, respectively. Distances in Å.

XeF_4_ is the most difficult fluoride of xenon to synthesize [126], and consequently, there are few examples of X-ray structures containing the XeF_4_ molecule in the database. They are represented in Figure 17, and all of them exhibit similar NgB π–hole interactions, which are established along the F–Xe–F bisector; this is in good agreement with the MEP surface analysis. In the X-ray structures of XeF_4_ (ICSD-27467 [127]), the XeF_2_·XeF_4_ adduct (ICSD-18128 [128]) and the [XeF_5_CrF_5_]_4_∙XeF_4_ adduct (ICSD-71592 [129], the XeF_4_ forms two equivalent Xe⋯F NgB interactions with the adjacent XeF_4_, XeF_2_, and CrF_6_ molecules (see Figure 17a–c). In contrast to a large number of XeF_2_ coordination compounds, metal complexes with XeF_4_ as ligands are rare due to the low basicity of XeF_4_. One selected example is represented in Figure 17d [130], where it is coordinated to the magnesium ion and, concurrently, forms a single NgB interaction with the nearby AsF_6_^−^ anion.

#### 2.4.3. X-Ray Structures of XeF_6_

**Figure 18:**
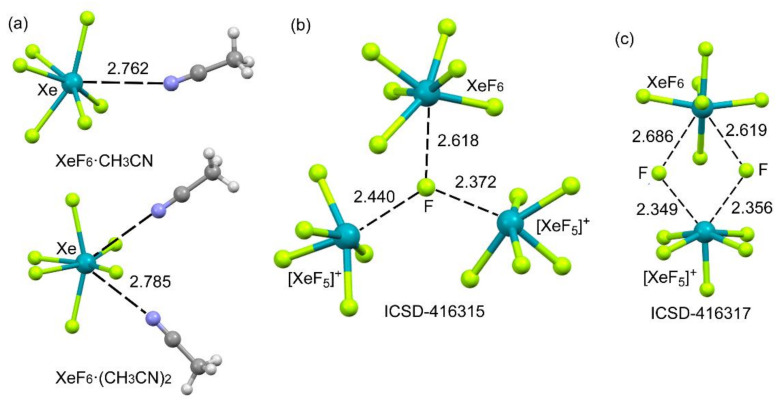
Partial views of the X-ray structures corresponding to XeF_6_·CH_3_CN adducts (**a**) and ICSD reference codes ICSD-416315 (**b**, m*C*_32_ phase) and ICSD-416317 (**c**, m*P*_32_ phase). Distances in Å.

Two adducts of XeF_6_ with acetonitrile were synthesized and characterized by single-crystal X-ray analysis in 2015 [131], which are represented in Figure 18a. These are the only available structures in the literature where the NgB interaction involves nitrogen as an electron donor and XeF_6_ as the Lewis base. Xe⋯N NgB interactions are more abundant in other Xe(VI) molecules such as those commented above for XeO_3_ and adducts of the F_2_Xe=O [132] molecule. The adducts geometry confirms that the XeF_6_ moieties are not octahedral, thus facilitating the approximation of the lone pair of acetonitrile to the Xe atom via the octahedral face where the σ–hole is located. 

It has been experimentally demonstrated that xenon hexafluoride may exist in six different polymorphs depending on the temperature [133]. The polymorphs that are stable at higher temperatures have been represented in Figure 18b,c. One of both (ICSD-416317) is formed by three (XeF_5_^+^F^−^) units and one XeF_6_ molecule. A partial view of its X-ray structure is depicted in Figure 18c where two fluoride anions connect the XeF_5_^+^ and XeF_6_ moieties though four Xe⋯F contacts. The NgBs involving the XeF_5_^+^ are shorter than those involving the neutral XeF_6_ due to the electrostatic attraction between the counter-ions. The other form of XeF_6_ (ICSD-416315) stable at high temperature was synthesized by sublimation of the other one. In this structure (see Figure 18b), the fluoride anion establishes three Xe⋯F contacts: one with the XeF_6_ unit, and two with the XeF_5_^+^ cations. The NgB distances in both polymorphs are similar, and the geometry of the XeF_6_ unit is approximately *C*_3v_ in the ICSD-416315 structure.

## 3. Concluding Remarks

This short review highlights the importance of π,σ–hole interactions involving xenon in XeO_3_ and XeF_n_ adducts. They are directional and comparable in strength to other noncovalent interactions where heavy atoms of groups 14–17 act as Lewis acids. The term noble gas bonding (NgB) refers to this interaction that has been studied in detail by several theoretical investigations. Moreover, the cooperativity of NgBs with other interactions such as hydrogen, halogen, chalcogen, pnicogen, and alkali bonds and anion/lone pair⋯π are commented. 

The X-ray structures selected in this short review highlight xenon’s ability as a π,σ–hole donor atom and provide experimental support to the importance of directional NgB in the solid state. The NgB dictates the crystal packing of XeO_3_ adducts by forming directional interactions with a variety of electron-rich atoms (O, N, F, Cl, Br). Moreover, there is a significant number of X-ray structures of XeF_2_, XeF_4_, and XeF_6_ adducts in the ICSD where noncovalent Xe⋯F contacts govern the crystal packing of these fascinating inorganic solids.

## Figures and Tables

**Table 1:** Interaction energies and equilibrium distances (ΔE in kcal/mol and d in Å) taken from ref. [81].

Complex	Δ*E*	d
**XeO_3_⋯NCCH_3_**	−9.5	3.142
**XeO_3_⋯NH_3_**	−9.0	2.779
**XeO_3_⋯Cl^−^**	−37.2	2.784
**XeO_3_⋯Br^−^**	−32.6	2.983
